# Low Pretreatment CD4^+^:CD8^+^ T Cell Ratios and CD39^+^CD73^+^CD19^+^ B Cell Proportions Are Associated with Improved Relapse-Free Survival in Head and Neck Squamous Cell Carcinoma

**DOI:** 10.3390/ijms241612538

**Published:** 2023-08-08

**Authors:** Ross J. Turner, Thomas V. Guy, Nicholas J. Geraghty, Ashleigh Splitt, Debbie Watson, Daniel Brungs, Martin G. Carolan, Andrew A. Miller, Jeremiah F. de Leon, Morteza Aghmesheh, Ronald Sluyter

**Affiliations:** 1 Molecular Horizons and School of Chemistry and Molecular Bioscience, University of Wollongong, Wollongong, NSW 2522, Australia; rturner@uow.edu.au (R.J.T.); geraghty@uow.edu.au (N.J.G.); dwatson@uow.edu.au (D.W.); 2 Illawarra Health and Medical Research Institute, Wollongong, NSW 2522, Australia; tguy1@mgh.harvard.edu; 3 Illawarra Cancer Care Centre, Wollongong Hospital, Wollongong, NSW 2500, Australia; ashleigh.splitt@health.nsw.gov.au (A.S.); daniel.brungs@health.nsw.gov.au (D.B.); martin.carolan@health.nsw.gov.au (M.G.C.); andrew.miller@health.nsw.gov.au (A.A.M.); morteza.aghmesheh@health.nsw.gov.au (M.A.); 4 Graduate School of Medicine, University of Wollongong, Wollongong, NSW 2522, Australia; 5 GenesisCare, St Vincent’s Clinic, Darlinghurst, NSW 2010, Australia; jeremy.deleon@genesiscare.com

**Keywords:** head and neck cancer, *ENTPD1*, *NT5E*, lymphocyte, human papillomavirus

## Abstract

The ectonucleotidases CD39 and CD73 are present on immune cells and play important roles in cancer progression by suppressing antitumour immunity. As such, CD39 and CD73 on peripheral blood mononuclear cells (PBMCs) are emerging as potential biomarkers to predict disease outcomes and treatment responses in cancer patients. This study aimed to examine T and B cells, including CD39 and CD73 expressing subsets, by flow cytometry in PBMCs from 28 patients with head and neck squamous cell carcinoma (HNSCC) and to assess the correlation with the treatment modality, human papillomavirus (HPV) status, and relapse-free survival (RFS). The PBMCs were examined pre-, mid-, and post-radiotherapy with concurrent cisplatin chemotherapy or anti-epidermal growth factor receptor antibody (cetuximab) therapy. Combination radiotherapy caused changes to T and B cell populations, including CD39 and CD73 expressing subsets, but no such differences were observed between concurrent chemotherapy and cetuximab. Pretreatment PBMCs from HPV^+^ patients contained increased proportions of CD39^−^CD73^−^CD4^+^ T cells and reduced proportions of CD39^−/+^CD73^+^CD4^+^ T cells compared to the equivalent cells from HPV^−^ patients. Notably, the pretreatment CD4^+^:CD8^+^ T cell ratios and CD39^+^CD73^+^CD19^+^ B cell proportions below the respective cohort medians corresponded with an improved RFS. Collectively, this study supports the notion that CD39 and CD73 may contribute to disease outcomes in HNSCC patients and may assist as biomarkers, either alone or as part of immune signatures, in HNSCC. Further studies of CD39 and CD73 on PBMCs from larger cohorts of HNSCC patients are warranted.

## 1. Introduction

Head and neck squamous cell carcinoma (HNSCC) includes cancers that originate from the mucosal surface of the oral cavity, pharynx, or larynx [1]. HNSCC has a worldwide incidence of approximately 900,000 new cases per year [2]. In addition, patients are commonly diagnosed with a regionally advanced stage of the disease, contributing to a poor prognosis and high mortality [3]. Typically, HNSCC of the oral cavity is treated with surgery, followed by adjuvant radiotherapy or chemoradiotherapy, while HNSCC of the pharynx or larynx is typically treated with primary chemoradiotherapy [4]. Cisplatin is the gold standard concurrent radiosensitiser in HNSCC, with anti-epidermal growth factor receptor antibody (cetuximab) therapy being commonly used in cisplatin-ineligible patients [4]. HNSCC typically arises from genetic alterations induced by heavy tobacco and alcohol consumption; however, it can also arise through oncogenic transformation following human papillomavirus (HPV) infection [4]. HPV-positive (HPV^+^) and HPV-negative (HPV^−^) HNSCC are molecularly distinct [5] and display different immune profiles [6,7,8]. Notably, HPV^+^ HNSCC demonstrates improved therapeutic responses and clinical outcomes [8,9].

Due to the heterogeneity of HNSCC, clinical outcomes in this cancer are often unpredictable, highlighting the need to find new biomarkers or immune signatures that will better predict the treatment response or disease outcomes [10]. Immune signatures can generally be defined as clusters of genes or proteins, including cell surface receptors or cytokines, whose expression or lack thereof, describe specific characteristics of a given cancer or cancer subtype [11]. These immune signatures may be within the tumour microenvironment or peripheral blood, such as patterns in tumour infiltrating or circulating immune cells, respectively. The identification of immune signatures in blood samples are ideal as blood collection is minimally invasive, commonly performed in routine clinical testing, and compared to tissue biopsies is less susceptible to selection biases [12,13], although standardisation and clear reporting of methods remains necessary [14].

CD39 (ectonucleoside triphosphate diphosphohydrolase-1) and CD73 (ecto-5′-nucleotidase) are ectonucleotidases expressed on the cell surface of immune and other cell types. CD39 subsequently hydrolyses adenosine 5′-triphosphate (ATP) and adenosine 5′-diphosphate (ADP) to adenosine 5′-monophosphate (AMP) [15]. CD73 then hydrolyses AMP to adenosine [16]. The adenosine producing CD39/CD73 pathway has various roles in many disorders, including cancer [17], multiple sclerosis [18], aortic stenosis [19], graft-versus-host-disease [20], and AIDS [21]. Most notably in cancer, the generation of adenosine exerts immunosuppressive effects by activating adenosine receptors on both innate and adaptive immune cells to dampen immune responses, including antitumour immunity [22]. As such, CD39 and CD73 represent emerging targets for checkpoint inhibitors, as their blockade can potentially restore or enhance antitumour responses [17]. Currently there are at least six CD39 checkpoint inhibitors (ES014, ES002023, JS019, PUR001, SRF617, and TTX-030) and at least 14 CD73 checkpoint inhibitors (AB680, AK119, ATG-037, CPI-006, IBI325, INCA00186, IPH5301, JAB-BX102, MEDI9447 (or oleclumab), ORIC-533, PT199, quemliclustat, Sym024, and TJ004309) that are undergoing clinical testing in subjects with solid tumours, as monotherapy or in combination with immunotherapy, chemotherapy, and/or radiotherapy (clinicaltrials.gov; accessed on 17 May 23).

Given the established role of CD39 and CD73 in antitumour responses and their potential roles as therapeutic targets, these ectonucleotidases are emerging as promising biomarkers, either alone or as part of immune signatures. For example, low baseline proportions of PD-1^+^CD73^+^CD8^+^ T cells are associated with improved overall survival and clinical benefit to anti-PD-1 (nivolumab) immunotherapy in metastatic melanoma patients [23]. Another study revealed that proportions of CD39^+^CD4^+^ T cells were increased in chronic lymphocytic leukaemia patients and associated with the advanced stage of this disease [24]. CD39^+^CD8^+^ T cells in the peripheral blood have also been suggested as a useful noninvasive biomarker to predict prolonged progression-free survival and overall survival in advanced non-small-cell lung cancer treated with pembrolizumab or nivolumab [25].

This current study aimed to examine and compare T and B cells, including CD39 and CD73 expressing subsets, in peripheral blood mononuclear cells (PBMCs) obtained from HPV^+^ and HPV^−^ HNSCC patients prior to, during, and following radiotherapy with concurrent cisplatin chemotherapy and/or cetuximab. In summary, radiotherapy with concurrent cisplatin or cetuximab caused changes to the T and B cell populations, including CD39 and CD73 expressing subsets, with no differences observed between cisplatin and cetuximab. Pretreatment PBMCs from HPV^+^ patients contained increased proportions of CD39^−^CD73^−^CD4^+^ T cells and reduced proportions of CD39^−^CD73^+^CD4^+^ and CD39^+^CD73^+^CD4^+^ T cells compared to the equivalent cell subsets from HPV^−^ patients. Finally, pretreatment CD4^+^:CD8^+^ T cell ratios or CD39^+^CD73^+^CD19^+^ B cell proportions below their respective medians correlated with longer relapse-free survival (RFS).

## 2. Results

### 2.1. Combination Radiotherapy Alters the Proportions of T and B Cells, including CD39 and CD73 Expressing Subsets

The proportions of CD4^+^CD3^+^ and CD8^+^CD3^+^ T cells and CD19^+^ and CD5^+^CD19^+^ B cells were examined in PBMCs collected from 21 of 28 patients with HNSCC prior, during, and following combination radiotherapy (Table 1). Due to the COVID-19 pandemic, seven patients were excluded due to the absence of mid- or post-treatment samples. CD5^+^CD19^+^ B cells were assessed, as an earlier study revealed that proportions of this B cell subset increase post-chemoradiotherapy [26].

**Figure 1:**
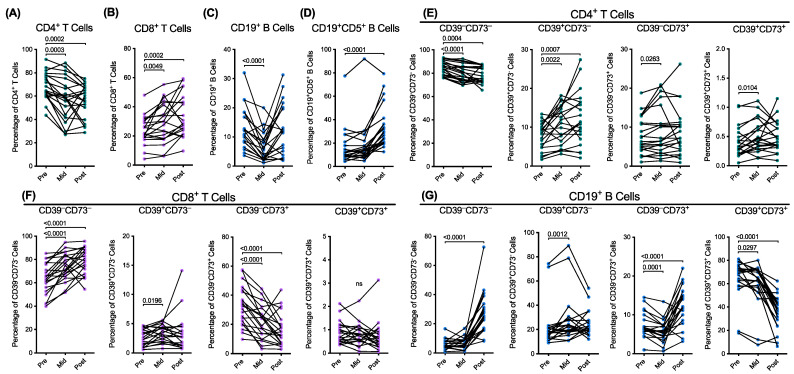
Combination radiotherapy alters the proportions of T and B cells, including CD39 and CD73 expressing subsets. (**A**–**G**) PBMCs from patients with HNSCC, at pre-, mid-, and post-treatment, were labelled with fluorochrome-conjugated mAbs and data acquired by flow cytometry. The live proportions of (**A**) CD4^+^ and (**B**) CD8^+^ T cells of the CD3^+^CD19^−^ T cells, (**C**) CD3^−^CD19^+^ B cells of the total lymphocytes, and (**D**) CD19^+^CD5^+^ B cells of the CD3^−^CD19^+^ B cells were determined by flow cytometry. The live proportions of CD39^+/−^CD73^+/−^ (**E**) CD4^+^ and (**F**) CD8^+^ T cells and (**G**) CD19^+^ B cells were also determined. The connected symbols represent individual donors (*n* = 21); *p* < 0.05 compared to the corresponding pretreatment PBMCs; paired two-tailed Student’s *t*-test or Wilcoxon matched-pairs signed rank test. ns represents not significant.

The mean proportions of the CD4^+^ and CD8^+^ T cells (amongst CD3^+^ T cells) pretreatment were 68.3 ± 11.7% (Figure 1A) and 23.9 ± 9.6%, respectively (Figure 1B). The proportions of the CD4^+^ T cells were significantly reduced by 15.2% (*p* = 0.0003) mid-treatment and by 17.4% (*p* = 0.0002) post-treatment (Figure 1A). Conversely, the CD8^+^ T cells were significantly increased 1.2-fold (*p* = 0.0049) mid-treatment and 1.4-fold (*p* = 0.0002) post-treatment (Figure 1B).

The proportions of CD19^+^ B cells (amongst lymphocytes) and CD5^+^ B cells (amongst CD19^+^ B cells) pretreatment were 13.2 ±7.1% and 16.6 ± 15.9%, respectively (Figure 1C,D). The proportions of CD19^+^ B cells were significantly reduced by 50.9% (*p* < 0.0001) mid-treatment but then recovered post-treatment to the pretreatment values (Figure 1C). The proportions of the CD5^+^ B cells pretreatment and mid-treatment were similar but significantly increased 2.0-fold post-treatment (*p* < 0.0001) (Figure 1D).

Next, the mean proportions of the T and B cell expressing combinations of CD39 and CD73 were assessed. Compared to the CD4^+^ T cells pretreatment, the proportions of the CD39^−^CD73^−^ cells were significantly reduced by 5.0% mid-treatment (*p* < 0.0001) and by 6.7% post-treatment (*p* = 0.0004) (Figure 1E). The proportions of the CD39^+^CD73^−^ cells were significantly increased 1.4-fold mid-treatment (*p* = 0.0022) and 1.6-fold post-treatment (*p* = 0.0007) (Figure 1E). The proportions of the CD39^−^CD73^+^ were significantly increased 1.2-fold mid-treatment (*p* = 0.0263) but were similar post-treatment (Figure 1E). The small proportions of CD39^+^CD73^+^ cells were significantly increased 1.2-fold mid-treatment (*p* = 0.0104) but were similar post-treatment (Figure 1E).

Compared to the CD8^+^ T cells pretreatment, the proportions of the CD39^−^CD73^−^ cells were significantly increased 1.2-fold mid-treatment (*p* < 0.0001) and 1.2-fold post-treatment (*p* < 0.0001) (Figure 1F). The proportions of the CD39^+^CD73^−^ cells significantly increased 1.2-fold mid-treatment (*p* = 0.0196) but were similar at post-treatment to pretreatment (Figure 1F). The CD39^−^CD73^+^ population was significantly reduced by 33.3% mid-treatment (*p* < 0.0001) and by 49.3% post-treatment (*p* < 0.0001) (Figure 1F). The small proportions of CD39^+^CD73^+^ cells remained unaltered over time (Figure 1F).

Compared to the CD19^+^ B cells pretreatment, the proportions of CD39^−^CD73^−^ cells were unaltered mid-treatment but significantly increased 4.6-fold post-treatment (*p* < 0.0001) (Figure 1G). The CD39^+^CD73^−^ population significantly increased 1.3-fold mid-treatment (*p* = 0.0012) but were similar post-treatment (Figure 1G). The proportion of the CD39^−^CD73^+^ cells was significantly reduced by 22.1% mid-treatment (*p* = 0.0001) but significantly increased 1.7-fold post-treatment (*p* < 0.0001) (Figure 1G). The CD39^+^CD73^+^ population was significantly reduced by 7.3% mid-treatment (*p* = 0.0297) and by 44.5% post-treatment (*p* < 0.0001) (Figure 1G). Due to the relatively low flow cytometric event numbers of the CD5^+^CD19^+^ B cells (results not shown), the proportions of the CD39^+/−^CD73^+/−^ cells in this population were not assessed.

Univariate analysis via the log-rank (Mantel–Cox) test revealed that the fold changes in the T or B cells, including CD39 and CD73 expressing subsets, between pre- and post-treatment were not associated with an altered RFS (results not shown).

### 2.2. Proportions of T and B Cells, Including Those Expressing Combinations of CD39 and CD73, Do Not Differ between Concurrent Cisplatin and Cetuximab

The current study afforded the opportunity to compare the impact of cisplatin chemotherapy or cetuximab in combination with radiotherapy on the proportions of T and B cells, as well as CD39 and CD73 expressing subsets. Therefore, the data from above (Figure 1) were stratified according to patients with HNSCC receiving concurrent chemotherapy or cetuximab. Patient 9 was excluded due to being treated with both cisplatin and cetuximab (with cisplatin changed to cetuximab during the treatment due to toxicities) (Table 1). Data are presented as fold changes from pretreatment to post-treatment. In this respect, there were no significant differences in the fold changes in CD4^+^ T cells (Figure S1A) or CD8^+^ T cells (Figure S1B) between cisplatin and cetuximab. Similarly, there were no significant difference in the fold changes in the CD19^+^ B cells (Figure S1C) or CD5^+^CD19^+^ B cells (Figure S1D) between treatment modalities. Moreover, there were no significant differences in the fold changes in the CD39^−^CD73^−^, CD39^+^CD73^−^, CD39^−^CD73^+^, or CD39^+^CD73^+^CD4^+^ T cells (Figure S1E), CD8^+^ T cells (Figure S1F) or CD19^+^ B cells (Figure S1G) between cisplatin and cetuximab. Moreover, the treatment modality was not associated with RFS (Figure S2A).

### 2.3. HPV Status Does Not Alter the Proportions of T or B Cells but Corresponds to Differences in the Pretreatment Proportions of CD73^+^CD4^+^ T Cells

**Figure 2:**
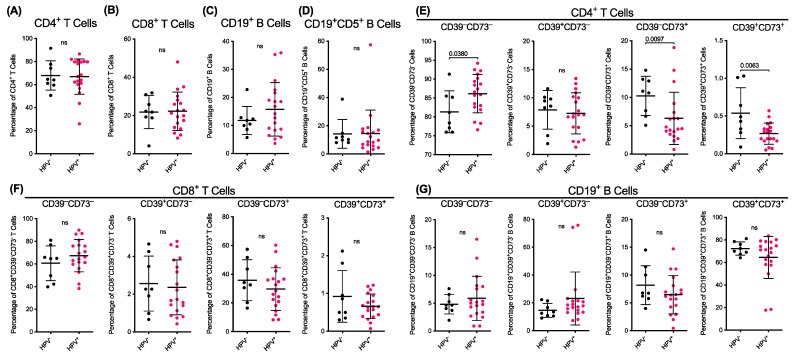
Human papillomavirus (HPV) status does not alter the proportions of T or B cells but corresponds to differences in the pretreatment proportions of CD73^+^CD4^+^ T cells. (**A**–**G**) HNSCC patients from Figure 2 were stratified by HPV status (HPV^−^, *n* = 8; or HPV^+^, *n* = 19) and presented as proportions of live cells. The data represent group means ± SD; the symbols represent individual donors; *p* < 0.05 indicates significance, ns means not significant; unpaired two-tailed Student’s *t*-test or Mann–Whitney U test.

Previous studies have demonstrated different immune profiles in patients with HPV^+^ HNSCC compared to HPV^−^ HNSCC [6,7,8]. Therefore, pretreatment data (Figure 1) were stratified according to HPV status. Patient 1 was excluded as the HPV status of the patient was unknown (Table 1). The analyses revealed that there were no significant differences in the proportions of the CD4^+^ T cells (Figure 2A), CD8^+^ T cells (Figure 2B), CD19^+^ B cells (Figure 2C) or CD5^+^CD19^+^ B cells (Figure 2D) between the HPV^−^ and HPV^+^ patients.

Amongst the CD4^+^ T cells, the proportion of CD39^−^CD73^−^ cells was 1.1-fold greater (*p* = 0.0380) in the HPV^+^ patients compared to those in the HPV^−^ patients (Figure 2E). There were no significant differences in the proportions of the CD39^+^CD73^−^ cells between the HPV^−^ and HPV^+^ patients (Figure 2E). Conversely, the proportions of the CD39^−^CD73^+^ and CD39^+^CD73^+^ cells in the HPV^+^ patients were significantly lower by 38.7% (*p* = 0.0097) and 50.3% (*p* = 0.0063) compared to the corresponding populations in the HPV^−^ patients, respectively (Figure 2E). Amongst the CD8^+^ T cells and CD19^+^ B cells, there were no significant differences in the proportions of the CD39^−^CD73^−^, CD39^+^CD73^−^, CD39^−^CD73^+^, or CD39^+^CD73^+^ cells between the HPV^−^ and HPV^+^ patients (Figure 2F,G). Despite the observed differences in CD39 and CD73 expressing CD4^+^ T cell subsets, HPV status was not associated with RFS (Figure S2B).

### 2.4. Low Pretreatment CD4^+^:CD8^+^ T Cell Ratios or CD39^+^CD73^+^CD19^+^ B Cell Proportions Are Associated with an Improved RFS

**Figure 3:**
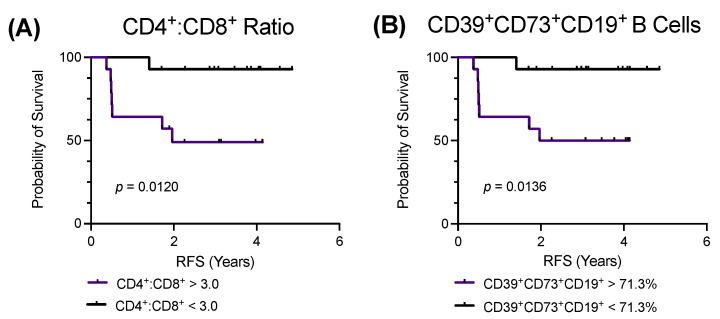
Low pretreatment CD4^+^:CD8^+^ T cell ratios or CD39^+^CD73^+^CD19^+^ B cell proportions are associated with improved relapse-free survival (RFS). RFS univariate analysis via the log-rank (Mantel–Cox), stratified according to high and low pretreatment (**A**) CD4^+^:CD8^+^ T cell ratios and (**B**) CD39^+^CD73^+^CD19^+^ B cell proportions to the median of 3.03 and 71.25%, respectively (*n* = 28). *p* < 0.05 indicates significance.

Finally, univariate analysis via the log-rank (Mantel–Cox) test was used to compare the RFS to the pretreatment proportions of T and B cells, including CD39 and CD73 expressing subsets. The pretreatment proportions of the CD4^+^ and CD8^+^ T cells, including CD39 and CD73 expressing subsets, were not associated with altered RFS (results not shown). However, patients with pretreatment CD4^+^:CD8^+^ T cell ratios below the cohort median ratio of 3.03 had a significantly improved RFS (*p* = 0.0120) (Figure 3A). The proportions of the pretreatment CD19^+^ and CD5^+^CD19^+^ B cells were not associated with an altered RFS (results not shown). Regarding the CD39 and CD73 expressing subsets, patients with pretreatment proportions of CD39^+^CD73^+^CD19^+^ B cells below the cohort median proportion of 71.3% had a significantly improved RFS (*p* = 0.0136) (Figure 3B). The proportions of other CD39 and CD73 expressing B cell subsets were not associated with altered RFS (results not shown).

## 3. Discussion

This study aimed to examine the proportion of T and B cell populations, including CD39 and CD73 expressing subsets, in PBMCs obtained from a small cohort of patients with HNSCC treated with radiotherapy, with reference to the impact of treatment modality, HPV status, and RFS. The results demonstrate that combination radiotherapy significantly impacted the CD4^+^ and CD8^+^ T cells and the CD19^+^ and CD5^+^CD19^+^ B cells, including subsets expressing combinations of CD39 and CD73. In contrast, there was no significant impact on these cell populations by cisplatin chemotherapy or cetuximab in combination with radiotherapy, suggesting that radiotherapy, rather than either systemic treatment, is the main cause of changes within the T and B cell proportions, including CD39 and CD73 expressing subsets. Compared to the HPV^−^ HNSCC patients, the HPV^+^ patients had increased proportions of CD39^−^CD73^−^CD4^+^ T cells and reduced proportions of CD39^−^CD73^+^CD4^+^ and CD39^+^CD73^+^CD4^+^ T cells. Notably, low pretreatment ratios of CD4^+^:CD8^+^ T cell or low proportions of CD39^+^CD73^+^CD19^+^ B cells were associated with an improved RFS.

Combination radiotherapy caused a reduction and increase in the proportion of CD4^+^ and CD8^+^ T cells, respectively, mid- and post-treatment. This is consistent with previous work demonstrating the impact of radiotherapy on T cells. Several studies on HNSCC have shown decreased proportions of CD4^+^ T cells and increased proportions of CD8^+^ T cells following chemoradiotherapy [27,28,29,30]. CD4^+^ T cells have important roles in antitumour immunity, which is largely attributed to the priming of CD8^+^ T cells to achieve the tumour killing effector function [31]. In addition to priming, CD4^+^ T cells can pair with other immune cells such as macrophages, eosinophils, and natural killer cells to mediate tumour clearance [31,32,33]. The observed increased proportions of CD8^+^ T cells mid- and post-treatment would theoretically have positive clinical implications due to increased tumour killing by these cells [34]; however, post-treatment proportions of CD8^+^ (or CD4^+^) T cells or their fold change were not associated with improved survival. Conversely, a study in oesophageal squamous cell carcinoma patients showed that increased CD8^+^ T cell ratios following chemoradiotherapy was associated with improved overall survival [30]. The observed increase in CD8^+^ T cells mid- and post-treatment may still be indicative of an antitumour response and expansion of tumour specific CD8^+^ T cells. Radiotherapy leads to liberation of tumour-associated antigens that stimulate maturation signals that promote migration of the antigen-presenting cells to the draining lymph nodes [35,36]. As such, T cell priming would dramatically increase in the draining lymph nodes, leading to a systemic antitumour response and an increase in the circulating tumour specific CD8^+^ T cells. However, due to the small number of patients in the current study who received adjuvant radiotherapy or chemoradiation, it was challenging to identify any differences in radiotherapy-induced tumour antigen release in the presence or absence of the primary tumour at this time.

Combination radiotherapy caused a transient reduction in the proportion of CD19^+^ B cells during treatment, with an increase in the proportion of CD5^+^CD19^+^ B cells post-treatment. There are inconsistent data regarding the impact of radiotherapy on the proportions of circulating CD19^+^ B cells and CD5^+^ B cells in HNSCC [26,27,29]. The decrease in B cells mid-treatment in the present study may have negative health impacts due to the potentially reduced humoral immunity in these patients during treatment [37]. The recovery of the CD19^+^ B cells is supported by the increase in CD5^+^CD19^+^ B cells post-treatment. CD5 is expressed by transitional and pre-naive B cells [38]; thus, the increase in the proportion of CD5^+^CD19^+^ B cells may represent the expansion of the B cell progenitors.

Combination radiotherapy caused changes to the T cells expressing combinations of CD39 and CD73. The increase in the proportion of the CD39^+^CD73^−^CD4^+^ T cells and corresponding decrease in the CD39^−^CD73^−^CD4^+^ T cells is currently, to the best of our knowledge, the first report of this pattern in any cancer type. CD39 is known to be highly expressed in T regulatory cells (Tregs) [39]. Thus, the increase in the CD39^+^CD73^−^CD4^+^ T cells in the present study may be due to the fact that the increased CD39^+^ Tregs are more resistant to radiotherapy compared to conventional CD4^+^ T cells [28]. If CD39^+^ Tregs are persisting during treatment, this could imply there is a greater suppression of antitumour activity occurring, a negative implication for treatment response [40]. Additionally, since CD39 is a proposed marker of CD4^+^ T cell activation [41,42] the increase in the CD39^+^CD73^−^CD4^+^ T cells may also represent activated CD4^+^ T cells. Regardless of the identity of these cells, this population was not associated with altered survival. Combination radiotherapy also caused a large decrease in the proportion of CD39^−^CD73^+^CD8^+^ T cells, with a corresponding increase in the CD39^−^CD73^−^CD8^+^ T cells. Naive CD8^+^ T cells preferentially express CD73, which is downregulated upon activation and differentiation into effector or memory CD8^+^ T cells [43]. This finding, along with the increase in CD8^+^ T cells, suggests there is an expansion of activated CD8^+^ T cells, as would be expected if these cells are becoming antigen-experienced following the release of tumour-associated antigens as a result of radiotherapy [35,36].

Combination radiotherapy decreased the proportion of the CD39^+^CD73^+^ B cells, consistent with other studies [26,29]. CD39^+^CD73^+^ B cells can promote immunosuppression by their combined ability to convert ATP and ADP to adenosine [29,44]. Moreover, radiotherapy can promote the release of ATP [45], leading to increased immunosuppression when converted to adenosine by CD39^+^CD73^+^ B cells [29,44]. Therefore, a reduced proportions of these cells during treatment may facilitate stronger antitumour immunity in cancer patients, leading to extended survival in cancer patients.

HPV^+^ patients displayed lower proportions of CD39^−/+^CD73^+^CD4^+^ T cells compared to those from HPV^−^ HNSCC patients. This finding is likely to reflect differences in the aetiology of these cancer types (viral versus carcinogen, respectively), with arguably the HPV^−^ HNSCC cohort displaying abnormally increased proportions of CD73^+^CD4^+^ T cells. In healthy individuals, CD73^+^CD4^+^ T cells represent ~5% of peripheral blood CD4^+^ T cells [46,47,48], a proportion similar to that observed for CD73^+^CD4^+^ T cells in the HPV^+^ HNSCC cohort. In healthy individuals, CD73^+^CD4^+^ T cells typically comprise effector memory cells with a T helper (Th) 17 and/or polyfunctional Th1.17 phenotypes [46,47]. Thus, the potential increase in CD39^−/+^CD73^+^CD4^+^ cells in HPV^−^ HNSCC patients may represent a heightened Th17 or Th1.17 inflammatory state compared to HPV^+^ HNSCC patients, possibly in response to different tumour aetiology and antigen expression. The observed increase in HPV^−^ HNSCC patients is unlikely to reflect differences in Tregs, as this population in people is largely considered to be devoid of cell-surface CD73 [46,47]. Regardless, these results suggest that differences in the proportions of CD39^−/+^CD73^+^CD4^+^ T cells may contribute to an immune signature to help distinguish between HPV^−^ and HPV^+^ HNSCC patients.

Arguably the most important finding of the current study was that low pretreatment ratios of CD4^+^:CD8^+^ T cell or low proportions of CD39^+^CD73^+^CD19^+^ B cells were associated with an improved RFS. The role of CD4^+^:CD8^+^ T cell ratios in cancer is controversial, varying across different cancer types [49,50,51,52,53,54,55,56]. Of note, a previous study also reported that a low peripheral blood CD4^+^:CD8^+^ T cell ratio was associated with longer progression-free survival and overall survival in a cohort of patients receiving palliative chemotherapy for initially unresectable recurrent/metastatic HNSCC [49]. To the best of our knowledge, the relationship of CD39^+^CD73^+^CD19^+^ B cells to survival is yet to be reported in any cancer type. However, as discussed above, the decreased pretreatment proportions of these cells may result in reduced immunosuppression to facilitate stronger antitumour immunity and extending survival in patients. Collectively, our results highlight the potential for predictive pretreatment immune biomarkers in HNSCC and provide a warrant for further evaluation of CD39 and CD73 as biomarkers or as part of immune signatures in HNSCC. Such studies would logically include examining CD39 and CD73 on other immune cells including T helper cell subsets, Tregs, and natural killer cells, which were not investigated in the current study.

In conclusion, this study demonstrates several changes in T and B cell populations in patients with HNSCC. As such these findings, based on a relatively small number of patients, warrant further examination in additional patients with HNSCC. Moreover, larger patient numbers and a longer time scale of disease, with further comparisons of other clinical parameters, such as stage of disease and disease-free and overall survival, may provide further opportunities to identify biomarkers or immune signatures relevant to disease type or progression.

## 4. Materials and Methods

### 4.1. Materials

Ficoll-Paque was from GE Healthcare (Uppsala, Sweden). RPMI-1640 medium (Life Technologies; Carlsbad, CA, USA) was prepared at the Illawarra Health and Medical Research Institute (Wollongong, Australia). Foetal calf serum (FCS) (heat-inactivated before use) was from Bovogen Biologicals (East Keilor, Australia). Dimethyl sulfoxide and trypan blue were from Sigma-Aldrich (St. Louis, MI, USA). Dulbecco’s phosphate-buffered saline (PBS) was from Life Technologies. The Zombie NIR Fixable Viability Kit was from BioLegend (San Diego, CA, USA). Fluorochrome-conjugated monoclonal antibodies (mAbs) (Table 2) were from BD Bioscience (San Jose, CA, USA).

### 4.2. Subjects and Sample Collection

All experiments with human blood were conducted in accordance with approval by the University of Wollongong and the Illawarra-Shoalhaven Local Health District Human Research Ethics Committee (Wollongong, Australia). Peripheral blood was collected from 28 patients with HNSCC (Table 1). We included patients with HNSCC who had curative treatment at Wollongong Hospital (Wollongong, Australia), which incorporated radiotherapy. Patients treated with either definitive radiation or primary surgery management and adjuvant radiotherapy were included. Patients received concurrent systemic treatments (cisplatin or cetuximab) as per standard care per the treating physicians. Generally, patients received cetuximab if deemed to have a medical contradiction to cisplatin. Blood was collected (between November 2017 and April 2021) by venepuncture into Vacutainer potassium EDTA tubes (BD Bioscience) and was stored at room temperature for up to 6 h before processing. PBMCs were isolated by Ficoll-Paque density gradient centrifugation and cryopreserved in RPMI-1640 medium containing 25% FCS and 10% dimethyl sulfoxide as described [57].

### 4.3. Immunophenotyping of PBMCs by Flow Cytometry

**Figure 4:**
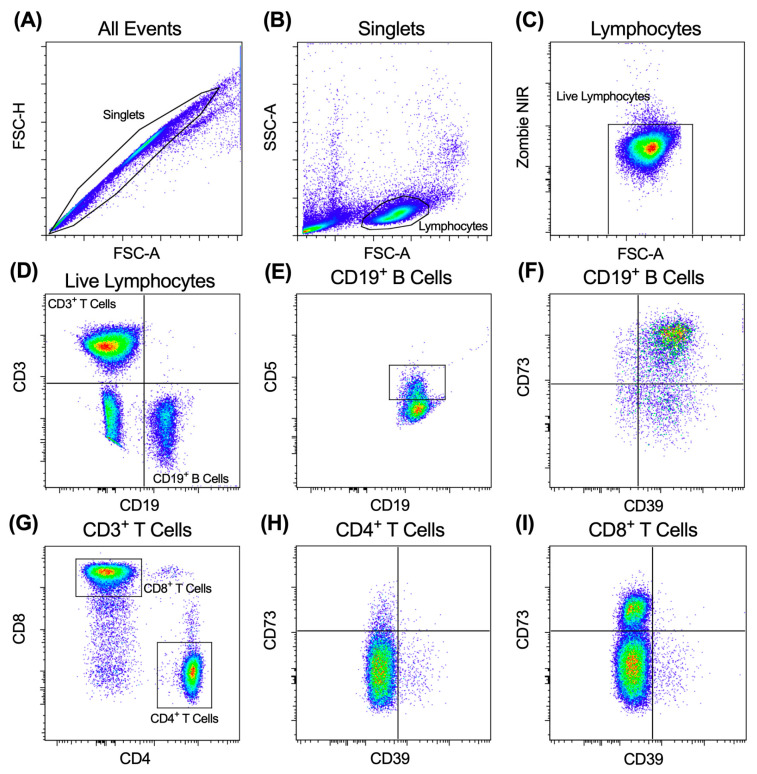
Gating strategy used for flow cytometric analyses of the HNSCC cohort. (**A**–**I**) PBMCs were labelled with fluorochrome-conjugated mAbs and data acquired by flow cytometry. Sequential flow cytometric gates were selected, as shown, to gate (**A**) single cells, (**B**) lymphocytes and (**C**) live (Zombie NIR^−^) lymphocytes, to determine the proportions of (**D**) CD3^−^CD19^+^ B cells and CD3^+^CD19^−^ T cells of total live lymphocytes. CD3^−^CD19^+^ B cells were gated to determine proportions of (**E**) CD5^+^CD19^+^ and (**F**) CD39^+/−^CD73^+/−^CD19^+^ B cells. CD3^+^CD19^−^ T cells were gated to determine the proportions of (**G**) CD4^+^ and CD8^+^ T cells. CD4^+^ and CD8^+^ T cells were gated to determine the proportions of (**H**) CD39^+/−^CD73^+/−^ CD4^+^ and (**I**) CD39^+/−^CD73^+/−^CD8^+^ T cells. One representative experiment of one subject is shown.

Cryopreserved PBMCs samples were thawed in a 37 °C water bath with constant swirling, washed with RPMI-1640 medium containing 10% FCS (RPMI-FCS) (300× *g*, 5 min, 21 °C), resuspended in RPMI-FCS, counted, and assessed for viability using trypan blue. The PBMCs were then washed with PBS (300× *g*, 3 min, 21 °C) and incubated with Zombie Dye NIR for 15 min at room temperature (protected from light). The cells were then washed with PBS containing 2% FCS and centrifuged (300× *g*, 3 min, 21 °C). The cells were then incubated with fluorochrome-conjugated mAbs (Table 2) for 15 min at room temperature (protected from light). The cells were finally washed with PBS (300× *g* 3 min, 21 °C), and a total of 5 × 10^4^ live lymphocytes were acquired using a BD Bioscience LSR Fortessa X-20 flow cytometer and BD FACSDiva software v8.0. Zombie dye NIR was detected using an excitation of 640 nm and a 780/60 nm band pass filter. Excitation and band pass filter wavelengths for each fluorochrome-conjugated mAb are listed in Table 2. The relative percentages of the lymphocyte subsets were analysed with FlowJo software v8.7.1 (TreeStar Inc., Ashland, OR, USA), using gating strategies as illustrated in Figure 4.

### 4.4. Statistics

Paired or unpaired data are represented as mean ± standard deviation (SD). The normality of the data was assessed using the Shapiro–Wilk test. The normally distributed data were compared using a paired or unpaired Student’s *t*-test (two-tailed). Non-normally distributed data were compared using a Wilcoxon matched-pairs test or a Mann–Whitney U test (two-tailed). Univariate analysis via the log-rank (Mantel–Cox) test was used to compare the various parameters to the RFS. All the statistical analyses and graphs were generated using Prism software version 8 (GraphPad, La Jolla, CA, USA). For all analyses, the differences were considered significant if *p* < 0.05.

## Figures and Tables

**Table 1:** Clinical characteristics of the HNSCC cohort.

Patient Code	Age (y)	Sex	Stage	HPV	Surgery	Rx	Radiosensitiser	Deceased
1	69	Male	IVA	U	-	Pri	Cet	Yes
2	68	Male	IVA	Neg	-	Pri	Cet	No
3	50	Male	IVA	Pos	-	Pri	Cx	No
4	56	Female	IVA	Neg	-	Pri	Cx	No
5	68	Male	III	Neg	-	Pri	Cx	Yes
6	58	Female	IVA	Pos	-	Pri	Cx	No
7	67	Male	III	Neg	-	Pri	Cet	No
8	64	Male	IVA	Pos	-	Pri	Cet	No
9	67	Male	II	Neg	-	Pri	Cx and Cet	No
10	76	Male	IVB	Pos	Pri	Adj	Cet	Yes
11	57	Male	III	Pos	-	Pri	Cet	Yes
12	66	Female	IVA	Neg	-	Pri	Cet	No
13	57	Male	IVB	Pos	-	Pri	Cx	Yes
14	60	Male	IVA	Pos	Pri	Adj	Cx	No
15	63	Male	II	Pos	-	Pri	Cet	Yes
16	48	Male	III	Pos	-	Pri	Cet	No
17	52	Male	IVA	Pos	-	Pri	Cx	No
18	77	Male	IVA	Pos	-	Pri	Cx	Yes
19	62	Male	IVA	Pos	Pri	Adj	Cx	No
20	59	Male	IVA	Pos	-	Pri	Cx	No
21	55	Male	IIIA	Pos	Pri	Adj	Cx	No
22	74	Male	IVA	Pos	-	Pri	Cx	No
23	59	Male	IVA	Pos	-	Pri	Cx	No
24	83	Male	IVA	Neg	-	Pri	Cx	No
25	56	Female	III	Neg	-	Pri	Cx	No
26	66	Male	IVA	Pos	-	Pri	Cx	No
27	68	Male	IVA	Pos	-	Pri	Cx	No
28	56	Male	IVA	Pos	-	Pri	Cx	No

Abbreviations: Adj, adjuvant; Con, concurrent; Cx, cisplatin chemotherapy; HPV, human papillomavirus; Cet, Cetuximab, anti-epidermal growth factor receptor antibody; Neg, negative; Pos, positive; Pri, primary; Rx, radiotherapy; U, unavailable; y, years.

**Table 2:** The mAbs used for staining and flow cytometric analyses of lymphocytes.

Marker	Clone	Catalogue Number	Fluorochrome	Excitation Wavelength	Bandpass Filter
CD3	UHCT1	563725	BV711	405	710/50
CD4	RPA-T4	555349	APC	640	670/30
CD5	UCHT2	555352	FITC	488	525/50
CD8	RPA-T8	562428	BV421	405	450/50
CD19	HIB19	561295	PerCP-Cy5.5	488	695/40
CD39	TU66	555464	PE	561	586/15
CD73	AD2	561258	PE-Cy7	561	780/60

Abbreviations: APC, allophycocyanin; BV, Brilliant Violet; FITC, fluorescein isothiocyanate; PerCP-Cy5.5, peridinin-chlorophyll-protein-Cyanine5.5; PE, phycoerythrin; PE-Cy7, phycoerythrin-cyanine.

## Data Availability

The data that support the findings of this study are available from the corresponding author upon reasonable request.

## Supplementary Materials

The following supporting information can be downloaded at: https://www.mdpi.com/article/10.3390/ijms241612538/s1.
