# Supplementary material for: Low Pretreatment CD4+:CD8+ T Cell Ratios and CD39+CD73+CD19+ B Cell Proportions Are Associated with Improved Relapse-Free Survival in Head and Neck Squamous Cell Carcinoma
Source: Int J Mol Sci. 2023 Aug 8;24(16):12538. doi: 10.3390/ijms241612538 (PMC10454544; doi:10.3390/ijms241612538)
Supplement: Supplementary file 1 [file ijms-24-12538-s001.zip › ijms-2524113-supplementary.pdf]

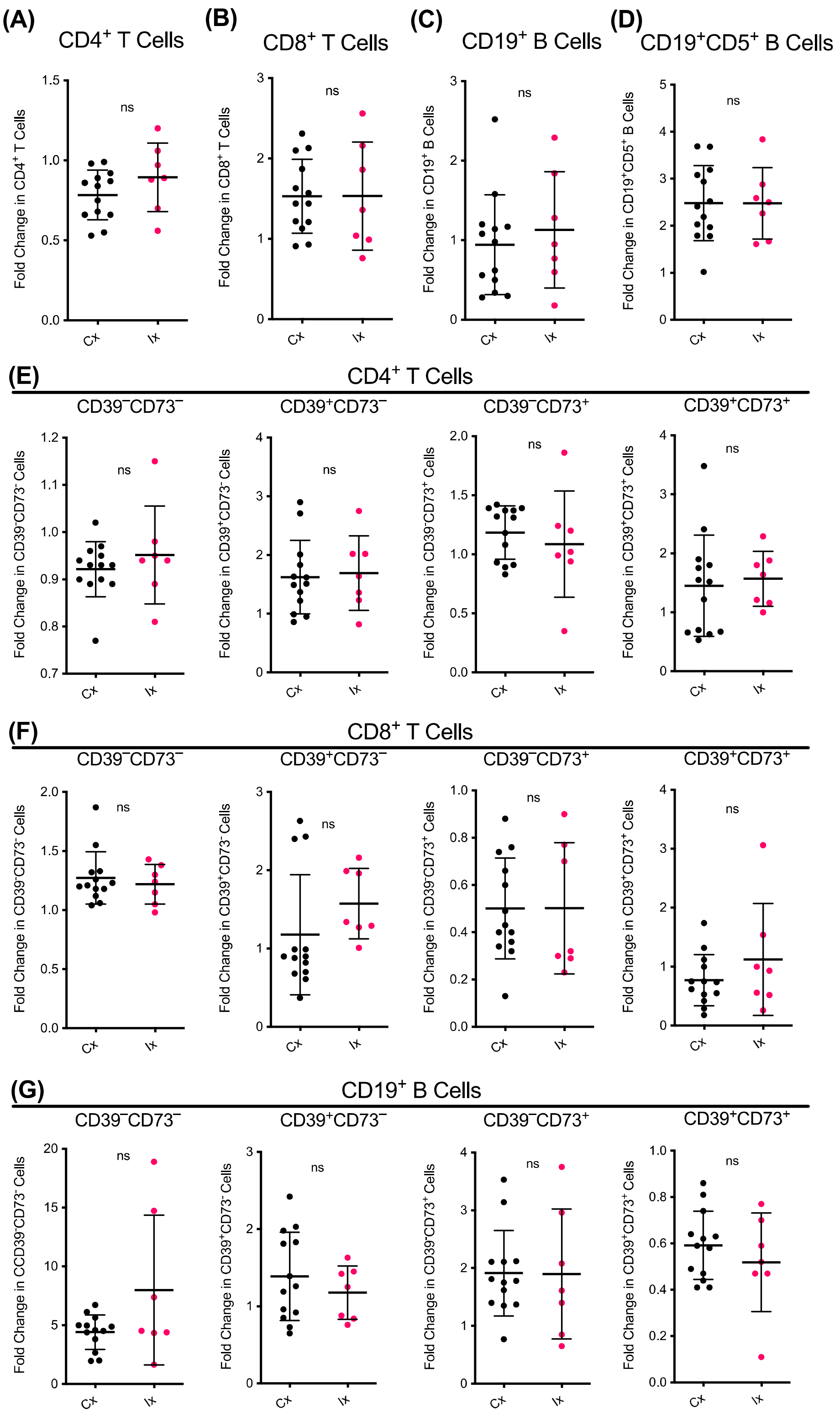

Figure S1. Proportions of T and B cells including those expressing combinations of CD39 and CD73 do not differ between concurrent chemotherapy (Cx) or Cetuximab (Ix). (**A–G**) HNSCC patients from Figure 2 were stratified by treatment (concurrent chemotherapy, $n = 13$; or Cetuximab, $n = 7$) and cell responses presented as fold change from pre-treatment to post-treatment. Data represents groups means ± SD; symbols represent individual donors; ns represents not significant; unpaired two-tailed Student's *t*-test or Mann-Whitney U test.

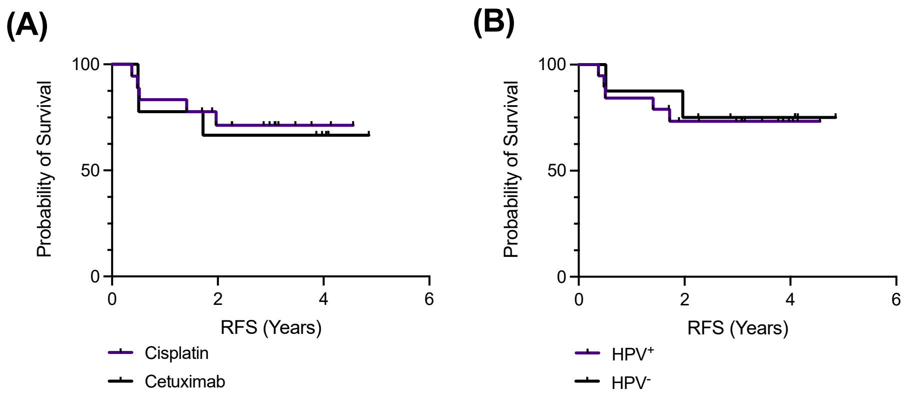

Figure S2. Treatment modality and human papillomavirus (HPV) status are not associated relapse free survival (RFS). RFS univariate analysis via the log-rank (Mantel–Cox), stratified according to (**A**) treatment modality (n = 27) and (**B**) HPV status (n = 27). No significant differences between groups.
